# Strategies for improving adipose-derived stem cells for tissue regeneration

**DOI:** 10.1093/burnst/tkac028

**Published:** 2022-08-16

**Authors:** Xin Yuan, Li Li, Haofan Liu, Jing Luo, Yongchao Zhao, Cheng Pan, Xue Zhang, Yuwen Chen, Maling Gou

**Affiliations:** State Key Laboratory of Biotherapy and Cancer Center, West China Hospital, Sichuan University, Chengdu, 610041, China; Department of Plastic and Burn Surgery, West China Hospital, Sichuan University, Chengdu, 610041, China; State Key Laboratory of Biotherapy and Cancer Center, West China Hospital, Sichuan University, Chengdu, 610041, China; State Key Laboratory of Biotherapy and Cancer Center, West China Hospital, Sichuan University, Chengdu, 610041, China; State Key Laboratory of Biotherapy and Cancer Center, West China Hospital, Sichuan University, Chengdu, 610041, China; State Key Laboratory of Biotherapy and Cancer Center, West China Hospital, Sichuan University, Chengdu, 610041, China; Department of Plastic and Burn Surgery, West China Hospital, Sichuan University, Chengdu, 610041, China; State Key Laboratory of Biotherapy and Cancer Center, West China Hospital, Sichuan University, Chengdu, 610041, China; State Key Laboratory of Biotherapy and Cancer Center, West China Hospital, Sichuan University, Chengdu, 610041, China; State Key Laboratory of Biotherapy and Cancer Center, West China Hospital, Sichuan University, Chengdu, 610041, China

**Keywords:** Adipose-derived stem cells, Tissue regeneration, Stem cell therapy

## Abstract

Adipose-derived stem cells (ADSCs) have promising applications in tissue regeneration. Currently, there are only a few ADSC products that have been approved for clinical use. The clinical application of ADSCs still faces many challenges. Here, we review emerging strategies to improve the therapeutic efficacy of ADSCs in tissue regeneration. First, a great quantity of cells is often needed for the stem cell therapies, which requires the advanced cell expansion technologies. In addition cell-derived products are also required for the development of ‘cell-free’ therapies to overcome the drawbacks of cell-based therapies. Second, it is necessary to strengthen the regenerative functions of ADSCs, including viability, differentiation and paracrine ability, for the tissue repair and regeneration required for different physiological and pathophysiological conditions. Third, poor delivery efficiency also restricts the therapeutic effect of ADSCs. Effective methods to improve cell delivery include alleviating harsh microenvironments, enhancing targeting ability and prolonging cell retention. Moreover, we also point out some critical issues about the sources, effectiveness and safety of ADSCs. With these advanced strategies to improve the therapeutic efficacy of ADSCs, ADSC-based treatment holds great promise for clinical applications in tissue regeneration.

Highlights

- ADSCs have great potential in tissue regeneration but the clinical translation of ADSCs remains a challenge.
- Strategies to improve the efficacy of ADSCs for tissue regeneration are reviewed.
- Critical issues about the sources, effectiveness and safety of ADSCs are raised for consideration.

## Background

Among various mesenchymal stem cells (MSCs), adipose-derived stem cells (ADSCs) are derived from adipose tissue and they have wide application potential for tissue repair and regeneration. Zuk *et al*. isolated a population of stem cells from human adipose tissue through liposuction and confirmed their stemness [1]. Adipose tissue provides an abundant autologous source of ADSCs and diverse preparation methods result in different ADSC-containing products. Generally, adipose tissue is harvested in a micro-invasive way through Coleman liposuction [2] or lipectomy. After a combination of mechanical emulsification, enzymatic digestion with collagenase and centrifugation, cells from fat tissue are concentrated and resuspended as the stromal vascular fraction (SVF) [3,4]. Next, the SVF is seeded in flasks and nonadherent cells are discarded after a certain period of time. Afterwards, cells are expanded in accordance with conventional culture standards [5,6]. In 2013, the International Federation for Adipose Therapeutics and Science and the International Society for Cellular Therapy made a joint statement that ADSCs share surface characteristics with MSCs, including positivity for cluster of differentiation (CD) 90, CD73, CD105 and CD44, and negativity for CD45 and CD31. Furthermore, ADSCs retain surface marker CD36 and lack CD106, which distinguishes them from bone-marrow-derived MSCs [7]. Different anatomical sites do not alter the biological properties or multipotency of ADSCs no matter whether they are obtained from visceral, omental or subcutaneous fat pads [8].

**Figure 1.**
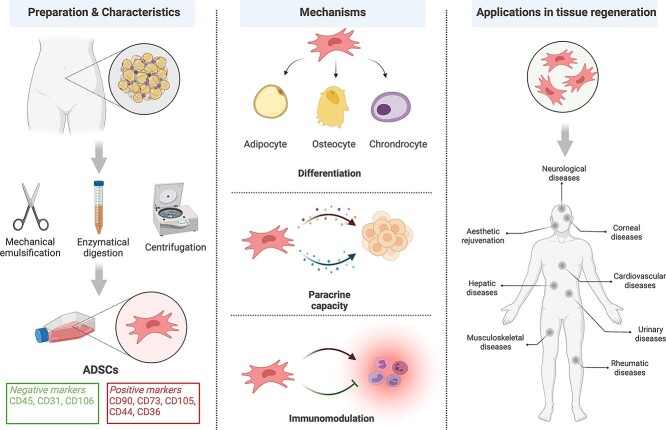
Preparation, characteristics, mechanisms and applications of adipose-derived stem cells (ADSCs) (created with BioRender.com)

Furthermore, ADSCs are one of the most favored ‘seed cells’ in regenerative medicine nowadays because of their ability to promote tissue regeneration via several mechanisms. First, ADSCs have multidirectional differentiation potential. In addition to osteogenic [9], chondrogenic [10] and adipogenic [11] differentiation potential, ADSCs are also capable of differentiating into hepatocytes [12,13], endothelial cells [14,15], smooth muscle cells [16,17], skeletal muscle cells [18], myocardial cells [19,20], Schwann cells [21] and pancreatic acinar-like cells [22]. ADSCs can also be reprogrammed into induced pluripotent stem cells (iPSCs) [23], whether they are derived from either human or mouse [24,25]. The reprogramming time for mouse and human ADSCs into iPSCs averages 1.5 weeks and 2.5 weeks, respectively, and their induction efficiency is higher than that of other types of cells [26]. The ability of ADSCs to differentiate into multilineage cells implies that ADSCs might replace damaged resident cells and benefit a wounded organ through differentiation. Another growing opinion insists that the healing competency of ADSCs in tissue regeneration should be ascribed to their paracrine capacity [27,28]. Angiogenic factors, growth and trophic factors, chemokines, pro-inflammatory cytokines, anti-inflammatory cytokines and other cytokines secreted by ADSCs [29], are dominant in tissue repair and play different roles during sequential wound healing processes. Therefore, we can prolong the paracrine period, extend the paracrine profile and strengthen the secretion of selected cytokines to reinforce the healing effect of ADSCs. Besides, ADSCs also have immunomodulatory functions. Both pro-inflammatory and anti-inflammatory effects of ADSCs have been reported. It is suggested that the stimulation of specific Toll-like receptors may lead to the polarization of MSCs into either pro-inflammatory or anti-inflammatory phenotypes, resulting in different immunomodulatory capacities under various pathophysiological conditions [30,31]. Therefore, stem cell therapy has been used in tissue regeneration mostly due to their anti-inflammatory ability for alleviating excessive inflammation in acute injuries and chronic inflammatory disorders. It is found that ADSC treatment can reduce inflammatory infiltration, impair Type 1 T helper (Th1)-driven inflammatory responses, promote macrophage polarization, downregulate the production of various inflammatory mediators and induce anti-inflammatory interleukin (IL) 10-secreting regulatory T cells [32–36]. Consequently, ADSC treatment can relieve symptoms of inflammatory bowel diseases, chronic obstructive pulmonary disease associated with cigarette smoking, crescentic glomerulonephritis, rheumatoid arthritis etc. [32–36]. Apart from the direct application of ADSCs, macrophages cultured with conditioned medium (CM) of ADSCs showed an immunosuppressive secretory phenotype, and therapeutic injection of these altered macrophages could also exert a protective effect against colitis [37]. ADSCs can also suppress the proliferation of lymphocytes, especially T cells [38], and prolong the survival of skin allografts *in vivo* [39]. These reports imply their low immunogenicity and suggest that they are well tolerated [40], which may enable the wide and safe application of ADSCs. Collectively, the abundant sources, low immunogenicity and ease of accessibility of ASDCs enable their broad application, and their capacity for multilineage differentiation, paracrine capacity and immunomodulation support their contributions to tissue repair (Figure 1).

Because of the above-mentioned advantages and characteristics, ADSCs have become an attractive tool for regenerative cell-therapies. Around 1000 MSC-related clinical trials and >400 clinical trials involving ADSCs are currently listed on ClinicalTrails.gov. The therapeutic potential of ADSCs has boosted numerous studies on musculoskeletal [41], cardiovascular [42–44], rheumatic [45], urinary [46–49], hepatic [50,51], corneal [52] and neurological diseases [53–55] as well as aesthetic rejuvenation [56] etc. (Figure 1). In addition to promoting tissue repair directly, adipocytes generated from patient-derived ADSCs carry genetic variation cues and help screen personalized antidiabetic drugs [57]. However, MSCs, as the mainstay for stem cell-based therapy, have always been the subject of criticism, because only a few clinical trials have succeeded in proceeding into the Phase III/IV stage. Some meta-analyses have pointed out that there is no level-4 evidence supporting stem cell therapy for tendon disorders [58] or knee osteoarthritis [59]. Furthermore, only a short list of cord blood based cellular products have been approved by the Office of Tissues and Advanced Therapies (OTAT) of the US Food and Drug Administration (FDA) [60],whereas none of the ADSC-derived products has been licensed in the USA. So far, approved ADSC products include Anterogen’s Cuepistem from Korea and Alopisel from Europe, both of which are indicated for the treatment of complex perinal fistulae in patients with Crohn’s disease. Collectively, ADSCs have great potential in tissue regeneration but the clinical translation of ADSCs remains a challenge.

## Review

### Strategies for improving ADSCs for tissue regeneration

**Figure 2.**
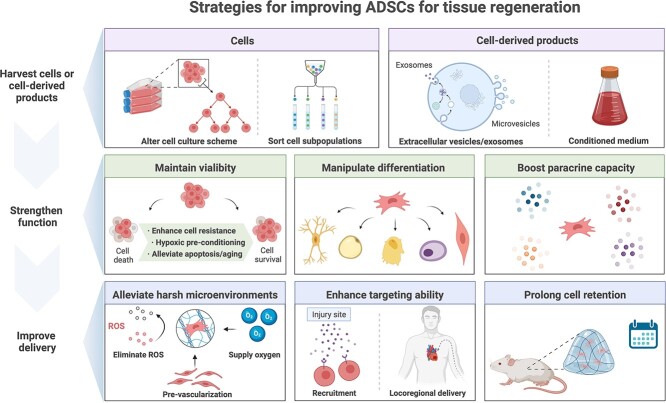
Strategies for improving adipose-derived stem cells (ADSCs) for tissue regeneration. *ROS* reactive oxygen species (created with BioRender.com)

It can be seen that, despite a surge in ADSC-based therapies, controversy persists and the optimal regimen for their clinical application remains elusive. Here, the needs and problems involved in the application of ADSC-based therapies are emphasized. Strategies to improve the efficacy of ADSCs for tissue regeneration are highlighted (Figure 2). First, a great quantity of cells is often needed for the stem cell therapies, which requires the advanced cell expansion technologies to maintain cell stemness during cell expansion for high-quality cell production. Meanwhile, sorting of cell subpopulations can offer specific therapeutic treatment. Besides, functional cell-derived products, such as extracellular vesicles/exosomes and conditioned medium, are also needed for the development of ‘cell-free’ therapies to overcome the drawbacks of cell-based therapies. Second, it is necessary to strengthen the regenerative functions of ADSCs, including viability, differentiation and paracrine ability, for the different physiological and pathophysiological conditions in tissue repair and regeneration. Third, poor delivery efficiency also restricts the therapeutic effect of ADSCs. Effective approaches to improving cell delivery include alleviating harsh microenvironments, enhancing targeting ability and prolonging cell retention. Finally, some crucial concerns on source, effectiveness and safety of ADSCs are raised for consideration when approaching better ADSC-based treatment. In summary, by aiming at critical points of ADSC application, diversified novel strategies can help in achieving the full potential of ADSC-based therapies.

### Harvest cells or cell-derived products

#### Cells

##### Alter cell culture scheme

The ever-growing use of stem cell-based regenerative medicine has been a challenge to the cell expansion technologies. A typical therapeutic dose of stem cells ranges from 10^6^ to 10^9^ cells [61–63]. Despite the rich resources of adipose tissue, the pressing need for large populations of stem cells necessitates simple, rapid and large-scale production. However, large-scale production may be accompanied by the possibility of abated cellular stemness, genetic and epigenetic mutations, phenotype transformation and functional property alteration due to rapid cell division. Hence, the cell culture scheme should be altered to meet the requirements of quality and quantity.

Cellular stemness can be preserved by designing scaffolds/substrate with a specialized nanostructure. For example, a ZnO nanorod array could provide a nanoscale surface and sustained release of Zn^2+^. Therefore, ADSCs cultured on a ZnO nanorod array were able to express Kruppel-like factor 4 and retain stemness genes and protein expression without inhibiting self-renewal and differentiation potential [64]. To prevent gradual stemness loss of cultured stem cells *in vitro*, it is necessary to avoid hyperoxia and intracellular reactive oxygen species (ROS) overload that is caused by frequently-used hydrophobic polystyrene flasks. Accordingly, a degradable zwitterionic hydrogel was developed to mitigate excessive ROS production within stem cells in response to hydrophobic substrates by inhibiting O_2_-related metabolism [65]. Another clickable zwitterionic starch-based hydrogel also contributed to the well-maintained stemness of encapsulated brown ADSCs against their spontaneous myocardial differentiation during expansion [66].

In addition, a favorable environment mimicking the *in vivo* stem cell niche can also help to maintain ADSC stemness and support stable and rapid proliferation. Usually, cells cultured within a 3D spheroid demonstrate better performance than those cultured on 2D culture plates because 3D culture can provide viable cell density and promote cell harvest. Besides, the 3D matrix is similar to the natural physiological milieu of ADSCs and is able to provide cell–cell and cell–extracellular matrix (ECM) mechanotransduction for improved proliferation and functionality. Construction of 3D stem cell aggregation via a chemical adhesion modification is advantageous to produce a potent pro-angiogenic secretome [67]. Formation of size-controllable ADSC spheroids induced by a nanowire surface could yield enhanced angiogenic potential [68].

Therefore, some ‘all-in-one’ platforms have emerged, such as hydrogels inspired by sandpaper [69] and lotus seedpod [70], which allowed integrated culture, harvest and delivery of ADSC spheroids. A thorough microsphere-based system, fabricated by digital light processing 3D printing technology, pulled together procedures including expansion, preservation and harvest. This system used cell-capsulated microspheres as functional units for ADSC delivery and ‘bottom-up’ tissue construction [71].

##### Sort cell subpopulations

ADSCs are known to be a heterogeneous population of cells that can be identified through a set of markers. The heterogeneity of stem cells results in divergent functionalities that may affect therapeutic potential. According to surface markers and other properties of stem cells, ADSCs can be further divided and enriched into subpopulations with specific functions and efficacy that can be used for effective and accurate treatment.

For example, purified CD105^+^ ADSCs was a worthwhile attempt to improve chondrogenic regeneration when seeded into a biodegradable 3D scaffold. [72]. CD105, i.e. endoglin, is a 180 kDa homodimeric transmembrane glycoprotein that acts as a coreceptor for transforming growth factor-β (TGF-β), which is a principal regulator during chondrogenesis [73]. Augmented cartilage regeneration could also be achieved by sorting and utilizing CD146^+^ ADSCs coupled with articular cartilage ECM in a rat osteochondral defect model. The therapeutic effect may benefit from the immune-modulating properties of CD146, a transmembrane glycoprotein and an adhesion molecule of the immunoglobulin superfamily [74].

#### Cell-derived products

##### Extracellular vesicles (EVs)/exosomes

Direct cell-based therapy often raises concerns about immune tolerance of allogenic cells, quality control of cell viability and stringent operation requirements, which drives the research into ‘cell-free’ therapies as viable alternatives.

Extracellular vesicles (EVs), as a novel acellular alternative for stem cell therapy, are described as cargoes containing selected proteins, lipids and nucleic acids [75]. The bilayer lipid membrane of EVs allows their fusion with target cells, and then microRNA (miRNA) and proteins released from vesicles mediate downstream signaling. It is a different process from direct paracrine effect which involves the transduction of extracellular signals via membrane receptors [76]. EVs contain not only conventional proteins but also tissue/cell type-specific contents, which reveal their source and specific utility. For example, EVs, obtained from ADSCs during differentiation towards white or beige adipocytes, favor white or beige adipogenic differentiation of ADSCs, respectively [77].

EVs isolated from supernatants of ADSCs via tangential-flow filtration have similar biological functions to ADSCs, such as immune-modulatory capacity. Intra-articular injection of ADSC-EVs significantly alleviated osteoarthritis and protected cartilage against degradation [78]. EVs effectively suppressed IL 1β-mediated expression of several inflammatory and catabolic factors, such as matrix metalloproteinase (MMP)-1, -3, -13 and a disintegrin and metalloproteinase with thrombospondin motifs (ADAMTS)-5, and EVs also strengthened chondrocyte viability and collagen deposition, thus shifting catabolic trends towards cartilage homeostasis [78]. Hypofunction of ultraviolet-damaged human dermal fibroblasts results in dermal inflammation, collagen loss and skin aging. Administration of ASDC-EVs could restore proliferation, migration and collagen synthesis of ultraviolet-irradiated human dermal fibroblasts and avoid aberrant matrix degradation [79]. Furthermore, after intrarenal ADSC-EVs infusion, pigs with metabolic syndrome and renal artery stenosis displayed improved performance of blood supply, glomerular filtration rate, medullary oxygenation and fibrosis rate. However, the renoprotective effect was attenuated with pre-silencing of IL-10 within EVs, which underlined the cargoes of anti-inflammatory factors in ADSC-EVs [80].

Apart from protein components, the therapeutic effect of ADSC-EVs could also be partly ascribable to nucleic acids within vesicles, such as miRNA. Taking advantage of osteoinductive miRNAs, immobilization of biotin-labeled ADSC-EVs on a biotin-doped polypyrrole titanium (Bio-Ppy-Ti) surface could enable titanium to be a promising metal graft for bone regeneration [81]. After internalization into osteoblasts, miRNA could enhance the expression of osteogenic genes and proteins, promote ECM mineralization and induce osteogenic differentiation.

In addition, many researchers have established ADSC-exosomes (exos) as an alternative approach for ADSC-based regenerative therapy. As determined by biogenesis, EVs can be divided into three main types, including exos, microvesicles and apoptotic bodies [82,83]. Exos, a large complex ranging from 50 to 100 nm in size, are secreted vesicles of endolysosomal origin, [76] while microvesicles bud from the plasma membrane [83]. Polydopamine-coating poly (lactic-co-glycolic acid) (PLGA/pDA) scaffolds immobilized with ADSC-exos demonstrated their potential for the repair of bone defects [84]. An injectable polypeptide-based hydrogel with a pH-responsive sustained release of ADSC-exos, could avoid the rapid clearance of ADSC-exos and achieve diabetic wound healing [85]. OxOBand, a porous cryogel supplemented with ADSC-exos, could also facilitate diabetic wound closure [86].

##### Conditioned medium

Utilization of ADSC-conditioned medium (CM) is another preferable ‘cell-free’ therapeutic. ADSC-CM contains almost the whole secretome of ADSCs and can reflect the regenerative potential that originate from their paracrine capacity. Compared with using EVs/exos, it is more economical, efficient, controllable and time-saving to employ ADSC-CM and utilization of ADSC-CM may avoid controversies arising from stem cell-based therapies. In addition, ADSC-CM treatment as a therapy involving a cocktail of factors is spatiotemporally quite similar to the physiological process. In contrast, it has been proven that individual delivery of growth factors may have drawbacks. For example, because of the mismatch between the *in vivo* kinetics of exogenous vascular endothelial growth factor (VEGF) and sequential angiogenesis demands, exogenous VEGF may result in a serious increase in vascular permeability [87].

It is reported that the healing potency of submucosal injection of ADSC-CM was equivalent to that of stem cell engraftment, in terms of proliferation, angiogenesis, immunomodulation and antioxidation [88]. Application of CM, including subcutaneous injection and topical smearing, achieved healing effects on a rat wound model through macrophage recruitment and macrophage polarization towards a pro-healing phenotype [89]. Instead of applying stem cells directly, simply treating endothelial cells with CM collected from an ADSCs sphere culture could promote capillary formation due to the increased level of VEGF production [68]. ADSCs cultured on polydopamine-modified bioceramic scaffolds showed enhanced paracrine function, the CM of which could promote angiogenesis, modulate macrophage phenotype and accelerate diabetic wound closure [90]. ADSC-CM could also protect lung epithelial cells against linear electrical injury and restore transcellular electrical resistance even in the presence of cigarette smoke extract, demonstrating the protective effect of ADSCs against cigarette-induced lung injury [33]. Additionally, a conceptual artificial stem cell spheroid (ASSP) was constructed by using PLGA and cell membranes of erythrocytes/platelets to load CM derived from ADSC spheroids. It was found that ASSP analogs restored blood perfusion against ischemic diseases, showing promise in the development of a controllable and applicable ‘cell-free’ ADSC-based therapy [67].

### Strengthen function

#### Maintain viability

Poor survival rate of ADSCs impedes their further application. How to maintain ADSC viability in a diseased microenvironment is a major challenge. Enhancing cell resistance, conducting hypoxic pre-conditioning and alleviating cell apoptosis and cell aging may be helpful.

Enhancing cell resistance to harsh environments is an option. Therefore, hydrogels immobilized with insulin-like growth factor 1C domain peptide improved the viability and angiogenic activity of encapsulated ADSCs *in vitro* as well as their survival and retention *in vivo*, thus achieving limb salvage for critical limb ischemia [91]. Additionally, melatonin could alleviate apoptosis, inflammation and oxidative stress of ADSCs by reducing the acetylation levels of p53, nuclear factor kappa-light-chain-enhancer of activated B cells (NF-κB) and forkhead box protein O1 (FoxO1) through upregulating sirtuin 1 (SIRT1). Therefore, melatonin exerted a protective effect against hypoxia/serum deprivation injury of ADSCs *in vitro* and showed an additional benefit in ADSC treatment of myocardial infarction [92]. The knockdown of prolyl hydroxylase domain protein 2 of ADSCs increased cell survival and insulin-like growth factor secretion and showed a cardioprotective effect, since prolyl hydroxylase domain protein 2 served as a sensor of cellular oxygen level and a regulator of two critical transcription factors, namely hypoxia-inducible factor (HIF) and NF-κB [93].

Moreover, hypoxic pre-conditioning can endow stem cells with enhanced adaptability to a hypoxic environment by up-regulation of the HIF-1 α pathway [94]. Pre-vascularized human MSC (hMSC) cell sheets that experienced hypoxic and angiogenic pre-conditioning through first-stage hypoxic culture conditions (2% O_2_) and second-stage co-culture with endothelial cells consequently displayed a superior therapeutic effect in terms of skin-graft take rate, tissue regeneration, cosmetic appearance and skin appendages preservation in comparison with untreated hMSC cell sheets [95].

Aging is also an issue of concern that may hinder the function of ADSCs. Delivery of lentivirus-containing myocardin and telomerase reverse transcriptase managed to promote myogenic differentiation and rejuvenate aged ADSCs, with therapeutic implication for ischemic diseases [96]. In addition, overexpressed CLOCK, a transcriptional activator regulating mammalian circadian rhythm, rejuvenated aged hMSCs *in vitro* and alleviated aging-related joint degeneration *in vivo* [97].

#### Manipulate differentiation

ADSCs can also differentiate into other cell types, the process of which can be guided and regulated by either intrinsic factors, like cell engineering, or extrinsic cues from the surrounding environment.

To achieve directed differentiation, overexpression of specific proteins in ADSCs by cell engineering is a common approach. For example, up-regulating of bone morphogenetic protein 2 (BMP2)/TGF-β3 reportedly augmented the osteogenic differentiation of ADSCs [98]. Modified by a hybrid baculovirus system to prolong and stabilize the expression of TGF-β3/BMP-6, rabbit ADSCs exhibited enhanced chondrogenesis without a tendency towards osteogenesis/hypertrophy [99,100]. In addition, ADSCs engineered through co-delivering the BMP2 gene for overexpression and the clustered regularly interspaced short palindromic repeats (CRISPR) interference system for Nog knockout, synergistically provoked osteogenic differentiation and induced healing of large calvarial bone defects [101]. Apart from those systems, lentivirus-mediated transfection of miR-135 into ADSCs could regulate osteogenic differentiation by down-regulating the downstream pathway of Homeobox A2. When combined with scaffolds, ADSCs transduced with miR-135 could increase the level of bone mineral density and trabecular number and effectively repair bone defects [102].

Extrinsic cues from the surrounding environment are also emphasized as stem cell fate regulators. The utilization of biomaterials contributes to multidimensional manipulation of stem cells and thus provides promising prospects for ADSC-based therapy in tissue engineering [103]. Stem cells sense and respond to the inherent properties of materials, including stiffness [104–107], geometry [108,109], adhesive ligands [106,110] and degradability [111], which conversely exert influence on stem cell morphology [110,112,113], cytoskeleton [114], mechanotransduction [114] and subsequent cell fate determination [115], demonstrating a dynamic cell–material interplay. Accordingly, advanced platforms have made it possible to orchestrate stem cell fate in a novel way, such as *in situ* magnetic control [116], high-throughput screening for beneficial cues [117] and monitoring [118,119], even at the single-cell level [118].

#### Boost paracrine capacity

It is widely believed that the paracrine capacity of ADSCs contributes to tissue regeneration, and how to boost their secretion to generate beneficial bioactive factors has always been a crucial concern in the field of tissue regeneration [33,88].

Cell engineering (i.e. transgene and gene editing), characterized by meticulous, directed, but complicated regulation, is one of the worthwhile attempts to evoke regenerative potential from an internal aspect. For example, engineered by Cas9 adeno-associated virus serotype 6 transduction, hMSCs overexpressed platelet-derived growth factor BB (PDGF-BB) and VEGFA accelerated the healing of diabetic wounds [120]. ADSC cell sheets functionalized by the CRISPRa system achieved potent peripheral nerve regeneration through overexpression of multiple neurotrophic factors [121].

Furthermore, external factors, including different biomedical, chemical and mechanical cues have been employed to stimulate ADSCs. Some strategies are straightforward and provide the needed stimulus directly. For example, tethering nanoparticles loaded with tumor necrosis factor α (TNFα) to the ADSC surface enhanced the secretion of pro-healing factors and achieved considerable blood perfusion and muscular restoration in hindlimb ischemia. The therapeutic effect of nanostimulators was comparable to that of preconditioned ADSCs, which may serve as a time-saving alternative to cell therapy [122]. There are also indirect, comprehensive methods, like structuring the microenvironment in favor of secretion. Formation of cell aggregates, including sheets [95,121,123], spheroids [67–70], fiberoids [124], microgels [125,126] etc., and implantation of cells within biocompatible scaffolds [89,90,127] could enhance regional cell density and modulate cell–cell and cell–material interaction, thus enhancing the paracrine effect of ADSCs [128]. For instance, in response to β-galactosidase, a specific hydrogel released nitric oxide and effectively activated the VEGF/VEGF receptor-2 pathway of ADSCs, thus triggering secretion of VEGF and stromal cell-derived factor (SDF)-1α for myocardial regeneration [129].

### Improve delivery

#### Alleviate harsh microenvironments

One of the major hurdles in ADSC-based therapy for ischemic tissue repair is the harsh microenvironment, characterized by a high level of ROS, insufficient oxygenation, poor blood perfusion, excessive inflammation etc. [130]. Previously, maintaining cell activity was mentioned to improve cell survival. It is also feasible to reduce adverse factors and provide beneficial factors to change a harsh environment.

One of the common strategies is to eliminate ROS within the damaged microenvironment. More specifically, ROS may damage the adhesion molecules of ADSCs, including integrin αV and β1, p-fak and p-src, as well as intercellular adhesion molecule 1 and vascular cell adhesion molecule 1 ligands on cardiomyocytes for stem cell homing and integration. It is reported that injectable chitosan hydrogel could eliminate excessive ROS, recover adhesive molecules of ADSCs and increase the level of SDF-1 for ADSC homing, thereby contributing to myocardial repair [131]. Another strategy took advantage of the anti-oxidative properties of fullerenol nanoparticles within an alginate hydrogel to reduce superoxide anion and hydroxyl radicals, thus showing a potent cytoprotective effect. ADSCs embedded in such an antioxidative hydrogel presented improved therapeutic efficiency for myocardial infarction via activation of extracellular signal-regulated kinase and p38 as well as inhibition of c-Jun N-terminal kinases [132].

*In vitro* pre-vascularization measures may be beneficial for implanted stem cells. A damaged microenvironment and insufficient transport of nutrients may lead to the necrosis of engrafted ADSCs and hinder regenerative efficacy, especially for cells implanted within large-sized constructs. Therefore, pre-vascularization, i.e. formation of microvascular networks within constructs prior to implantation, may help to accelerate inosculation with host microvasculature and shorten the duration of hypoxia [133]. An engineering scaffold pre-seeded with human umbilical vein endothelial cells (HUVECs) and ADSCs in a 1:1 ratio could synergistically form aligned vascular networks after 11-day culture, which could meet the increased need for nutrients and oxygen of a large tissue by accelerating surrounding perfusion into volumetric muscle loss defects [134]. In addition, a 3D-printed skin patch, pre-vascularized with endothelial progenitor cells together with ADSCs, could remarkably induce angiogenesis and wound healing [135]. Furthermore, inspired by the multilineage differentiation potential of ADSCs, endothelial-induced ADSCs and osteogenically induced ADSCs were used for the chronological pre-vascularization of scaffolds to repair critical-size bone defects in rat femur [136].

Another way is to supply oxygen, since large volume materials may aggravate the hypoxia of newly formed tissue. For this reason, supplementation of perfluorotributylamine (PFTBA), an oxygen carrier with high oxygen solubility, within a hydrogel could enrich local oxygen supply and facilitate stem cell survival and tissue regeneration [137]. As less integration of stem cells at the infarcted site did not alter the therapeutic efficacy, it may be unnecessary to transplant cells at the injury site for regeneration [138]. Therefore, injecting stem cells into a site with sufficient blood supply rather than ischemic tissue may serve as an easy and direct treatment to bypass a harsh microenvironment.

#### Enhance targeting ability

Targeted administration is a preferred delivery method for ensuring the therapeutic potential and security of ADSCs because the majority of stem cells systemically injected become trapped in the capillary beds of lungs and the reticuloendothelial system of spleen and liver. Some studies suggested that although intravenously injected MSCs were embolized in the lungs, they could also be activated to release anti-inflammatory TNFα-induced protein 6, which partially contributed to the therapeutic effect of MSCs on myocardial infarction in mice. MSCs, which are not blood-resident cells, may be incompatible with human blood in varying degrees, thus impairing their safety and efficacy [139]. Therefore, targeted administration, including recruitment to the injury site and locoregional delivery, may improve the therapeutic potential and security of ADSCs.

To improve ADSC recruitment to the injury site, immobilization of target ligand on the cell surface is helpful for its targeted delivery and implantation. Surface functionalization of cells using heparin could avoid lung entrapment, increase accumulation and prolong retention of intravenously administered ADSCs in the liver, and thus further intensify the effect of ADSCs for the treatment of acute liver failure [140–142]. Encapsulation of ADSC-CM with functional, specific cell membrane also serves as an approach. Erythrocyte membrane coating can be used for prolonged blood circulation while platelet membranes are useful for targeting vascular injury [67,143,144]. Besides, upregulating expression of SDF-1 and some other injury-related chemokines after myocardial infarction could synergistically engage intravenous ADSCs to the infarcted site [145].

Additionally, locoregional delivery presents a more direct way to utilize ADSCs in close-quarters therapy, such as intra-arterial (coronary artery, hepatic artery, renal artery, carotid artery, femoral artery for the target organs, respectively), intra-portal, intra-cavity (nasal cavity, vitreous cavity, trachea and bronchus, cerebrospinal fluid cavity, articular cavity, abdominal cavity, urethra, vagina, digestive tract, etc.), within tissue/parenchyma (muscle) and *in situ* (cornea, skin, etc.) [146]. Advanced biomaterials and techniques can also help to achieve locoregional delivery. After injection into porcine fistula and gelling *in situ* at body temperature, thermoresponsive Pluronic F-127 gel managed to preserve ADSC-EVs in the fistula tract, leading to the desired healing results [147]. Moreover, the muscular defect could be restored by injection of ADSC-laden bioink and subsequent conformal scaffold formation by noninvasive *in vivo* 3D bioprinting, avoiding iatrogenic injury and surgical exposure [148]. Uniform administration of stem cells to skin wound area can also be achieved by in situ bioprinting [149] and cell spray devices [150].

#### Prolong cell retention

A noteworthy drawback of cell-based therapy is that administered cells tend to disperse to surrounding sites or interstitial space rather than to the intended tissues and/or organs, which may restrict the healing potential. Therefore, how to prolong ADSCs retention at the intended sites remains a crucial challenge for cell-based therapy.

One of the applicable approaches is to enhance the adhesive ability of ADSCs. Incorporation of integrin-specific short peptides into hydrogels, such as GFOGER (a specific amino acid sequence high affinity for α2β1 integrin), supported integrin-mediated stem cell adhesion and yielded potent survival and persistence of engrafted ADSCs [151]. In addition, because of the reversible Schiff base reaction between aldehydes and amino groups, the bioadhesive hydrogel enabled stable immobilization of ADSCs on a dynamic beating heart [152]. Moreover, ADSCs overexpressing N-cadherin displayed robust cell adhesion, migration, proliferation and paracrine capacity via β-catenin-dependent MMP-10/MMP-13/hepatocyte growth factor upregulation. Intramyocardial administration of modified ADSCs with increased retention and angiogenesis could protect cardiomyocytes against ischemic heart failure [153]. Administration in the form of cell aggregates, such as spheroids, sheets, fiberoids etc., could take advantage of inherent cell–cell connections and preserve adhesive molecules without the intervention of biomaterials or digestive enzymes, thus improving the retention of ADSCs at wound bed and injured site to prevent them being washed away [123,124,154–156].

Microgels encapsulating ADSCs also represent an alternative way to prolong cell retention and improve therapeutic potential. Microgels could promote cell–cell and cell–ECM interaction, enable high local cell-loading and avoid mechanical damage due to injection [157]. Through this microgel-assisted delivery, long-term retention of stem cells and prominent production of ECM and angiogenetic factors could be achieved [125,126]. Magnetic guidance could also augment the retention of MSCs incorporated with iron oxide nanoparticles (IONPs) at infarcted myocardium, as well as upregulate healing-related signaling pathways and promote the expression of growth factors, thus accelerating the reparative process [158].

### Critical issues on the sources, effectiveness and safety of ADSCs

Further understanding of the sources, effectiveness and safety of stem cells remains to be unveiled. First, as to the source of stem cells, autologous ADSCs are always a favorable choice. However, the inherent disease context of patients, such as systemic sclerosis [159], atherosclerosis [160], diabetes [161], obesity [162,163], aging [164] etc., might impair the reserve, secretory profiles and functional properties of ADSCs. Although serious adverse events on usage of allogenic ADSCs have rarely been reported so far, suggesting that cells from allogenic source are well tolerated [165–167], more evidence is needed to justify the clinical use of allogenic ADSCs. In addition, other than live-cell therapy, it has been highlighted that cellular debris from stem cells killed by freezing and thawing [168] and apoptotic cells along with their apoptotic bodies [169] may also serve as potential therapeutics.

Next comes the question of the effectiveness of stem cell therapy. Several aspects need to be emphasized including proper indication selection, standardization, cell dosage, route of administration, comparison with existing treatments and consideration of cost-effectiveness. The ultimate outcome of engrafted ADSCs, differentiation and integration, necrosis and apoptosis, or aberrant hyperplasia and even tumorigenesis, remains to be elucidated [170,171]. Revealing their *in vivo* fate helps researchers to manipulate *in vivo* behavior of ADSCs on demand, which may enable effective and safe ADSC-based therapies.

The most crucial issue, we believe, lies in safety concerns. High cell dose is often required for clinical remedies [172], which may increase the possibility of embolism and thrombosis through intravascular delivery [173]. In addition, long-term *in vitro* expansion may cause phenotype alternation and drive cell senescence [174]. In particular, utilization of cell-derived products and *in vitro* pretreatment may necessitate multiple complicated procedures and technologies. Some drawbacks of emerging technologies have also become a concern for researchers, including unexpected integration of retroviruses [175], off-target effects of the CRISPR-Cas system [176] and unpredictable toxicity of materials [177]. When administrated *in vivo*, stem cells may interact with the inherent inflammatory context of patients [162,163] and exert potential tumor-promoting effects [178]. Therefore, more caution and consideration are needed when ADSC-based therapy caters for cancer patients, e.g. ADSC-assisted breast reconstruction for postoperative breast cancer patients [179].

Furthermore, the thriving of ADSC-related research is accompanied by many challenges in commercialization and regulation. The unregulated commercial stem cell industry not only impedes the basic and clinical research of stem cell therapy but also jeopardizes the health and lives of patients [180]. The external chaos of the stem cell industry should not be an obstacle to its development. It is necessary to reasonably regulate and guide the development of stem cell research.

## Conclusions

ADSCs hold great promise for tissue regeneration, but translation of ADSC-based therapies to the clinic has been limited and requires further development of novel strategies. Strategies to improve ADSCs for tissue regeneration include harvesting cells or cell-derived products, strengthening function and improving delivery. Meanwhile, some critical issues about the sources, effectiveness and safety of ADSCs should also be taken into consideration before clinical application. Overall, by aiming at key points of the application process, advanced strategies are expected to overcome these obstacles and drive the development of ADSCs for tissue regeneration.

## Abbreviations

ADSCs: Adipose-derived stem cells; MSCs: Mesenchymal stem cells; SVF: stromal vascular fraction; CD: Cluster of differentiation; iPSCs: Induced pluripotent stem cells;CM: Conditioned medium; ROS: Reactive oxygen species; ECM: Extracellular matrix; TGF-β: Transforming growth factor-β; EVs: Extracellular vesicles; miRNA: microRNA; MMP: Matrix metalloproteinase; Exos: Exosomes; PLGA/pDA: Polydopamine-coating poly(lactic-co-glycolic acid); VEGF: Vascular endothelial growth factor; ASSP: Artificial stem cell spheroid; NF-κB: Nuclear factor kappa-light-chain-enhancer of activated B cells; HIF: Hypoxia-inducible factor; BMP: Bone morphogenetic protein; CRISPR: clustered regularly interspaced short palindromic repeats; TNFα: Tumor necrosis factor α; SDF: Stromal cell-derived factor.

## Funding

This work was supported by National Key Research and Development Program of China (2021YFF1200800, MLG), Sichuan Innovative Research Team Program for Young Scientists (2021JDTD0001, MLG), Post-Doctor Research Project, West China Hospital, Sichuan University (2020HXBH025, XY), 1·3·5 project for disciplines of excellence, West China Hospital, Sichuan University (ZYJC18017, MLG; ZYJC21079, CP).

## Conflict of interest

None declared.
